# 1021. Case Study. Beyond Standard Scales: Dynamic Pain Management in a Critically Ill Pediatric Burn Patient

**DOI:** 10.1093/jbcr/irag033.382

**Published:** 2026-04-06

**Authors:** Tinjia Hwang, Mini Thomas

**Affiliations:** UCI Health, Orange, CA; UCI Health, Orange, CA

## Abstract

**Patient Presentation (age range, injury details, relevant history):**

Assessing pain in pediatric burn patients is challenging, especially when the child is nonverbal, critically ill, and immobilized. This case highlights these challenges in a 5-year-old with extensive burns to 48.5% of the body, bilateral transradial amputations, bilateral femur fractures, partial facial excision, and prolonged mechanical ventilation requiring tracheostomy. The child’s condition precluded the use of standard pediatric pain scales reliant on facial expression, verbal communication, and motor response.

**Clinical Challenges:**

In the acute phase, the patient was comatose, sedated, and intubated. Tools such as the FLACC (Face, Legs, Activity, Cry, Consolability) and Wong-Baker FACES were ineffective due to immobility, facial injuries, and lack of responsiveness. The Critical-Care Pain Observation Tool (CPOT) was chosen as most applicable, though even it was limited, as only the ventilator compliance domain could be assessed consistently. With no single tool sufficient for this case, adjunctive strategies were used, including physiologic indicators (heart rate, blood pressure), anticipating painful procedures, and observing patterns in autonomic response. The child’s mother also played a vital role, helping the team identify subtle behavioral cues that guided treatment and comfort measures.

**Management Approach:**

Over several weeks, the patient was weaned from ventilation and sedation. As alertness improved, new nonverbal signals emerged, such as facial grimacing, leg retraction, and purposeful eye movements. These evolving cues prompted a transition to the FLACC scale, which offered a more behaviorally attuned framework for the patient’s developing responsiveness. Its broader observational domains allowed for more consistent and sensitive monitoring compared to earlier tools.

**Outcomes:**

In our retrospective review, we examined several sedation and pain assessment tools not routinely used or considered standard in our unit, seeking more accurate options for this complex case. Although not used at the time, the COMFORT scale may have added value during the early sedated phase. Rarely used outside specialized pediatric settings, it offers a structured way to interpret physiologic indicators (HR and BP) when behavior is difficult to observe.

**Lessons Learned:**

This case highlights the importance of a dynamic, individualized approach to pain assessment in critically ill, nonverbal pediatric patients, one that adapts not only to the clinical phase but also to the patient’s evolving ability to express discomfort. It also underscores the power of interdisciplinary collaboration: from nurses who recognized behavioral changes, to the medical team who modified pain protocols, and the patient’s.

family, whose familiarity with baseline behaviors proved invaluable for personalized care. The team’s collective insight made the pain more visible and treatable.

**Applicability to Practice:**

Ultimately, this case challenges the assumption that one tool fits all. It supports the need for flexible, patient-centered strategies that evolve with the child and clinical situation, as outlined in Table 1. It suggests empowering teams to choose from multiple pain scales. Future research may focus on developing a pediatric scale that addresses pain across all stages of critical illness, eliminating the need to use multiple tools. As pediatric burn care advances so must our tools and mindset for recognizing and relieving pain in our most critically ill and vulnerable patients.

